# Impact of axillary disease extent defined by baseline ^18^F-FDG PET/CT on the accuracy of axillary surgical staging after neoadjuvant systemic therapy in clinically node-positive breast cancer

**DOI:** 10.1016/j.breast.2026.104718

**Published:** 2026-01-31

**Authors:** Cornelis M. de Mooij, Janine M. Simons, Florien J.G. van Amstel, Cristina Mitea, Paul J. van Diest, Patty J. Nelemans, Felix M. Mottaghy, Carmen C. van der Pol, Ernest J.T. Luiten, Linetta B. Koppert, Marjolein L. Smidt, Thiemo J.A. van Nijnatten, REFINE Study Group, E.G. Boerma, M. Boskamp, E.M.J. Brouwers-Kuyper, C.M.E. Contant, L. de Beer, C. de Monyé, A.W.F. du Mée, H.J. Heijmans, S. Ho-Han, F. Hulsebosch, A. Jager, J.A.J. Janssen, B.L.R. Kam, W. Kelder, T.M.A.L. Klem, K.P. Koopmans, M.B.I. Lobbes, M.B.E. Menke-Pluijmers, P. Sars, L.H.M. Smit, E. van Haaren, D. van Klaveren, J. Veltman, C. Verhoef, W.J. Vles

**Affiliations:** l GROW – Research Institute for Oncology and Developmental Biology, Maastricht University, Maastricht, the Netherlands; m Department of Internal Medicine, Maastricht University Medical Center+, Maastricht, the Netherlands; n Department of Surgical Oncology, Wilhelmina Hospital, Assen, the Netherlands; o Department of Radiology, Albert Schweitzer Hospital, Dordrecht, the Netherlands; p Department of Surgical Oncology, Maasstad Hospital, Rotterdam, the Netherlands; q Department of Radiology, Martini Hospital, Groningen, the Netherlands; r Department of Radiology and Nuclear Medicine, Erasmus Medical Center, Rotterdam, the Netherlands; s Department of Radiology, Amphia Hospital, Breda, the Netherlands; t Department of Surgical Oncology, Hospital Group Twente, Breast Clinic Oost-Nederland, Hengelo, the Netherlands; u Department of Nuclear Medicine, Albert Schweitzer Hospital, Dordrecht, the Netherlands; v Department of Radiology, Franciscus Gasthuis & Vlietland, Schiedam, the Netherlands; w Department of Medical Oncology, Erasmus Medical Center Cancer Institute, Rotterdam, the Netherlands; x Department of Nuclear Medicine, Ikazia Hospital, Rotterdam, the Netherlands; y Department of Nuclear Medicine, Diakonessenhuis, Utrecht, the Netherlands; z Department of Surgical Oncology, Martini Hospital, Groningen, the Netherlands; aa Department of Surgical Oncology, Franciscus Gasthuis & Vlietland, Schiedam, the Netherlands; ab Department of Nuclear Medicine, Martini Hospital, Groningen, the Netherlands; ac Department of Medical Imaging, Zuyderland Medical Center, Sittard-Geleen, the Netherlands; ad Department of Surgical Oncology, Albert Schweitzer Hospital, Dordrecht, the Netherlands; ae Department of Surgical Oncology, Bravis Hospital, Roosendaal, the Netherlands; af Department of Surgical Oncology, Treant Zorggroep Hospital, Hoogeveen, the Netherlands; ag Department of Surgical Oncology, Zuyderland Medical Center, Sittard, the Netherlands; ah Department of Public Health, Erasmus Medical Center, Rotterdam, the Netherlands; ai Department of Radiology, Hospital Group Twente, Breast Clinic Oost-Nederland, Hengelo, the Netherlands; aj Department of Surgical Oncology, Erasmus Medical Center Cancer Institute, Rotterdam, the Netherlands; ak Department of Surgical Oncology, Ikazia Hospital, Rotterdam, the Netherlands; a Department of Surgery, Maastricht University Medical Center+, Maastricht, the Netherlands; b Department of Radiology and Nuclear Medicine, Maastricht University Medical Center+, Maastricht, the Netherlands; c GROW – Research Institute for Oncology and Developmental Biology, Maastricht University, Maastricht, the Netherlands; d Department of Radiotherapy, Erasmus MC Cancer Institute, University Medical Center Rotterdam, Rotterdam, the Netherlands; e Department of Pathology, University Medical Center Utrecht, Utrecht, the Netherlands; f Department of Epidemiology, Maastricht University, Maastricht, the Netherlands; g Department of Nuclear Medicine, RWTH Aachen University Hospital, Aachen, Germany; h Department of Surgical Oncology, Alrijne Hospital, Leiderdorp, the Netherlands; i Department of Surgery, Amphia Hospital Breda, Breda, the Netherlands; j Department of Surgery, College of Medicine and Health Sciences, United Arab Emirates University, Abu Dhabi Emirate, Al Ain, United Arab Emirates; k Department of Surgical Oncology, Erasmus MC Cancer Institute, University Medical Center Rotterdam, Rotterdam, the Netherlands

**Keywords:** Breast cancer, Axillary lymph nodes, Neoadjuvant chemotherapy, ^18^F-FDG PET/CT

## Abstract

**Background:**

In clinically node-positive patients, sentinel lymph node biopsy (SLNB), marking axillary lymph node with radioactive iodine seed (MARI), and combined SLNB/MARI (RISAS-procedure) can replace axillary lymph node dissection (ALND) after neoadjuvant systemic therapy. Surgical staging outcome can be combined with baseline axillary disease on ^18^F-FDG PET/CT. This study assessed whether baseline axillary disease on ^18^F-FDG PET/CT affects the accuracy of staging-procedures. Second, when staging-procedures detected residual disease, it was assessed whether baseline axillary disease on ^18^F-FDG PET/CT affected the probability of remaining positive nodes at completion ALND (cALND).

**Method:**

Included were patients with baseline ^18^F-FDG PET/CT within the RISAStrial (NCT02800317). Patients underwent the RISAS-procedure followed by cALND. False negative rates were stratified by limited or advanced baseline axillary disease (1-3 vs. ≥4 hypermetabolic lymph nodes). When staging-procedures detected residual disease, the probability of remaining positive nodes at cALND was stratified by baseline axillary disease.

**Results:**

Of 185 patients, 116 had limited and 69 had advanced baseline axillary disease. Staging-procedures had higher accuracy in limited than advanced baseline axillary disease. When the RISAS-procedure detected residual disease, the probability of remaining positive nodes at cALND was lower in limited than advanced baseline axillary disease (44.9% vs. 91.5%,p < .001). When SLNB or MARI detected residual disease, the probability of remaining positive nodes at cALND was >88.4%, irrespective of baseline axillary disease.

**Conclusion:**

Staging-procedures had higher accuracy in patients with limited than advanced axillary disease on baseline ^18^F-FDG PET/CT. When staging-procedures detected residual disease, the probability of remaining positive nodes at cALND remained high.

## Highlights

• Surgical staging was followed by completion axillary lymph node dissection.
• Accuracy of surgical staging was higher with 1-3 hypermetabolic nodes at baseline.
• Detection of disease at surgical staging indicated high risk of remaining disease.
• Risk of disease after surgical staging is crucial to guide adjuvant treatment.

## 1 Introduction

Today neoadjuvant systemic therapy (NST) is administered in an increasing number of clinically node-positive (cN+) breast cancer patients [1,2]. Axillary pathologic complete response (pCR) is achieved in approximately 37% of the patients after NST [[3], [4], [5]]. Irrespective of axillary response to NST, axillary lymph node dissection (ALND) has long been the routine approach for cN + breast cancer. However, ALND and its associated comorbidity is not expected to benefit patients with an axillary pCR [6]. In an attempt to identify axillary pCR after NST without the need to perform ALND, several less invasive surgical staging procedures were proposed including the sentinel lymph node biopsy (SLNB) [7], excision of a pretreatment-marked positive axillary lymph node [8], and procedures that combine excision of a pretreatment-marked positive lymph node with SLNB (e.g., targeted axillary dissection) [9]. Currently, different markers are being used in TAD-procedures as highlighted by a systematic review by de Wild et al. [8]. In case the SLNB is combined with marking axillary lymph nodes with radioactive iodine seeds (MARI), this is called the RISAS-procedure [10].

These less invasive surgical staging procedures aim to reduce surgical morbidity but carry the risk of a false negative outcome, which results in leaving behind therapy-resistant residual disease in unresected axillary lymph nodes and may give rise to later recurrence [4]. Therefore, it is important to aim for the highest diagnostic accuracy regarding detection of axillary lymph nodes with residual disease in order to guide adjuvant axillary treatment appropriately [11]. Furthermore, in case less invasive surgical staging procedures detect and remove residual disease, estimates of the probability of leaving behind unresected axillary lymph nodes with (therapy-resistant) residual disease are important as, in these cases, there is still a need for adjuvant axillary treatment [11,12].

To improve adjuvant axillary treatment protocols, it has been proposed to not only consider the outcome of less invasive surgical staging procedures after NST but also the number of hypermetabolic axillary lymph node metastases (ALNMs) on baseline ^18^F-fluorodeoxyglucose (FDG) PET/CT (prior to NST). Van der Noordaa et al. developed a treatment protocol that combines axillary disease extent (i.e., 1-3 vs. ≥4 hypermetabolic ALNMs) on baseline ^18^F-FDG PET/CT in combination with the outcome of the MARI procedure after NST to de-escalate adjuvant axillary treatment in cN + patients [13]. However, it is yet unclear to what extent the number of hypermetabolic ALNMs at baseline ^18^F-FDG PET/CT affects the diagnostic accuracy of currently used less invasive surgical staging procedures. Moreover, in those patients with residual disease detected and removed by less invasive surgical staging, it is yet unclear whether the number of hypermetabolic ALNMs at baseline ^18^F-FDG PET/CT affects the risk of (therapy-resistant) residual disease being present in axillary lymph nodes that were not resected with the less invasive surgical staging procedure.

The first aim of this study was to assess whether axillary disease extent on baseline ^18^F-FDG PET/CT affects the diagnostic accuracy of the SLNB, MARI, and RISAS-procedure. The second aim was to evaluate whether axillary disease extent on baseline ^18^F-FDG PET/CT affects the probability that after detection and removal of residual disease during staging by SLNB, MARI, and RISAS-procedure there are remaining positive lymph nodes according to completion ALND (cALND).

## 2 Methods

### 2.1 Study design and population

This retrospective study used data from patients who participated in the prospective multicentre noninferiority RISAS trial (NST02800317; METC number 2016-412). Due to the retrospective analyses of this study, the necessity to obtain written informed consent was waived by the local medical ethics committee at each participating institute (reference number: 2020-2422).

This study included RISAS patients who underwent baseline ^18^F-FDG PET/CT. A detailed description of the RISAS trial is given in the RISAS study protocol [14] and in the publication of the primary results of the RISAS trial [10]. Patients participating in the RISAS trial were female patients aged ≥18 years with pathologically proven cN + breast cancer (cT1-4 and cN1, cN2, or cN3b). Axillary lymph node metastases were confirmed with either fine needle aspiration cytology or core needle biopsy prior to NST.

### 2.2 [^18^F]fluorodeoxyglucose PET/CT imaging

Patients underwent a whole-body ^18^F-FDG PET/CT exam with a standard acquisition protocol according to the protocol of the involved institution to exclude distant metastasis. In all institutions, baseline ^18^F-FDG PET/CT was performed according to the prevailing procedure guidelines of the European Association of Nuclear Medicine [[15], [16], [17]]. Patients had to fast for at least 4 h before ^18^F-FDG administration. Afterwards, blood glucose levels were checked to ensure levels below 11 mmol/l and subsequently the intravenous ^18^F-FDG injection was administered. A whole-body ^18^F-FDG PET/CT exam was acquired after a resting period of 45-60 min.

### 2.3 Neoadjuvant systemic therapy

Recommendations for NST regimens during the RISAS trial were based on prevailing Dutch national breast cancer guidelines [18] and generally involved anthracycline- and/or taxane-based regimens. Patients with HER2-positive breast cancer received additional targeted therapy (trastuzumab alone or trastuzumab and pertuzumab).

### 2.4 The RISAS-procedure

After NST, patients underwent the RISAS-procedure followed by cALND within a single surgical procedure. The RISAS-procedure is a combination of the SLNB and MARI. Prior to NST, ultrasound-guided placement of a radioactive iodine seed within a pathologically proven lymph node was performed. After NST, the SLNB was performed for which a dual-tracer technique was recommended but not obligated. In the same setting, the axillary lymph node marked with the radioactive iodine seed was detected with a gamma probe and subsequently excised. Suspicious and palpable non-sentinel lymph nodes were removed at the discretion of the surgeon.

### 2.5 Histopathology

During the RISAS trial, the histopathological results from cALND were used as reference. The results were reported separately for axillary lymph nodes retrieved at the SLNB, MARI, and cALND. All excised axillary lymph nodes were stained with haematoxylin and eosin. On-site use of immunohistochemistry was not mandatory. All axillary lymph nodes of the RISAS-procedure that were considered negative after on-site evaluation were centrally reviewed by an expert breast pathologist (P.J.v.D.) [10]. Axillary pCR was defined as the absence of any residual disease, including isolated tumour cells.

### 2.6 Baseline ^18^F-FDG PET/CT evaluation

**Fig. 1:**
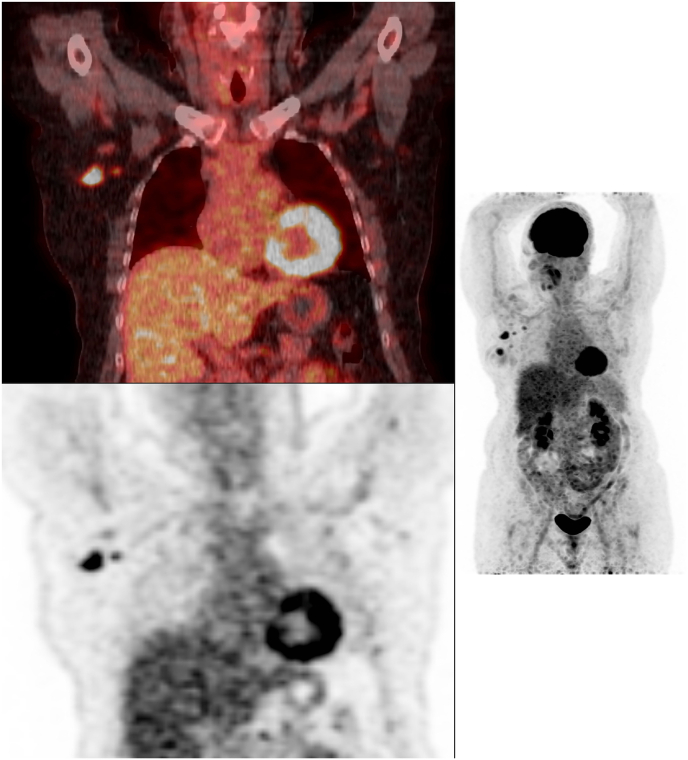
Baseline ^18^F-FDG PET/CT images of a RISAS patient with advanced baseline axillary disease (5 hypermetabolic ALNMs) with axillary residual disease at surgical staging. *FDG, fluorodeoxyglucose; ALNM, axillary lymph node metastases*.

For the current study, all baseline ^18^F-FDG PET/CT exams were centrally reviewed on a dedicated commercially available workstation (Syngo.via 6.4, Siemens Healthcare, Erlangen, Germany). The baseline ^18^F-FDG PET/CT exams were reviewed to determine the number of hypermetabolic ALNMs, performed by a nuclear radiologist (T.J.A.v.N.) with 4 years of clinical experience in ^18^F-FDG PET/CT and breast imaging (reviewer 1). Each axillary lymph node visible on ^18^F-FDG PET/CT was scored using a four-point confidence scale (0, similar to surrounding axillary lymph nodes; 1, slightly more intense than other axillary lymph nodes; 2, moderately intense; 3, very intense) [19]. Patients were classified as having limited (1-3 hypermetabolic ALNMs) or advanced (≥4 hypermetabolic ALNMs) baseline axillary disease (Fig. 1). The number of hypermetabolic ALNMs from the initial report were compared with the number of hypermetabolic ALNMs reported by reviewer 1. In case of a discrepancy in the number of hypermetabolic ALNM between the initial report and reviewer 1, a nuclear medicine physician (C.M.) with nine years of clinical experience in ^18^F-FDG PET/CT imaging (reviewer 2) reviewed the discrepant exams.

### 2.7 Statistical analysis

To evaluate whether axillary disease extent on baseline ^18^F-FDG PET/CT affects diagnostic accuracy, patients were categorized into two subgroups of patients with limited and advanced baseline axillary disease (1-3 vs. ≥4 hypermetabolic ALNMs). Both subgroups were compared with respect to the false negative rate (FNR) and negative predictive value (NPV) of the SLNB, MARI, and RISAS-procedure and the probability of remaining positive lymph nodes at cALND after detection and removal of residual disease during staging. The Chi square or Fisher's exact test was used for testing the difference in proportions between the two groups.

The FNR was defined as the number of false negative (FN) results divided by the total number of patients with axillary residual disease which is the sum of FN plus true positive (TP) results [FNR = FN/(FN + TP)]. The NPV was defined as the number of true negative (TN) results divided by the total number of patients with a negative test result according to the SLNB, MARI or RISAS-procedure [NPV = TN/(TN + FN)].

In the analyses, the total population consisted of patients in whom the concerning procedure was performed successfully (i.e. at least one lymph node was resected at surgical staging).

P-values <0.05 were considered to indicate statistical significance. Statistical analyses were performed with the Statistical Package for the Social Sciences (SPSS) software (Version 27, IBM, Armonk, New York, USA).

## 3 Results

### 3.1 Baseline and treatment characteristics

Of 185 included patients, 62.7% (116/185) had limited and 37.3% (69/185) had advanced axillary disease on baseline ^18^F-FDG PET/CT. The mean age was 52 years and mean breast tumour size was 40 mm. Overall, 29.7% (55/185) of the patients achieved axillary pCR. The axillary pCR rates did not differ significantly between patients with limited and advanced axillary disease on baseline ^18^F-FDG PET/CT (31.9% (37/116) vs. 26.1% (18/69), respectively, p = .403). Baseline and treatment characteristics of the study population are summarized in Table 1.

**Table 1:** Baseline and treatment characteristics.

Characteristic	Overall		Hypermetabolic ALNMs on baseline^18^F-FDG PET/CT			
			1-3 (n = 116)		≥4 (n = 69)	
**Age (years)**						
Mean (SD)	52	(11.1)	52	(11)	51	(11)
Median (range)	51	(22 – 76)	53	(27 – 76)	51	(22 – 76)
**Tumour size (mm)**						
Mean (SD)	40	(20)	38	(19)	44	(21)
Median (range)	36	(12 – 120)	33	(12 – 120)	40	(14 – 11)
**Clinical T-status**						
cTx	3	(1.6)	3	(2.6)	0	(0.0)
cT1	19	(10.3)	13	(11.2)	6	(8.7)
cT2	116	(62.7)	78	(67.2)	38	(55.1)
cT3	29	(15.7)	17	(14.7)	12	(17.4)
cT4	18	(9.7)	5	(4.3)	13	(18.8)
**Clinical N-status**						
cN1	130	(70.3)	107	(92.2)	23	(33.4)
cN2	39	(21.1)	4	(3.5)	35	(50.7)
cN3b	16	(8.6)	5	(4.3)	11	(15.9)
**ER**						
Negative	65	(35.1)	38	(32.8)	27	(39.1)
Positive	120	(64.9)	78	(67.2)	42	(60.9)
**PR**						
Negative	88	(47.6)	52	(44.8)	36	(52.2)
Positive	97	(52.4)	64	(55.2)	33	(47.8)
**HER2**						
Negative	127	(68.6)	79	(68.1)	48	(69.6)
Positive	58	(31.4)	37	(31.9)	21	(30.4)
**Molecular subtype**						
HR+/HER2-	86	(46.5)	56	(48.3)	30	(43.5)
HR+/HER2+	38	(20.5)	24	(20.7)	14	(20.3)
HR-/HER2+	20	(10.8)	13	(11.2)	7	(10.1)
HR-/HER2-	41	(22.2)	23	(19.8)	18	(26.1)
**Axillary response**						
RD	130	(70.3)	79	(68.1)	51	(73.9)
pCR	55	(29.7)	37	(31.9)	18	(26.1)

Values in parentheses are percentages unless indicated otherwise. ALNMs, axillary lymph node metastases; FDG, fluorodeoxyglucose; SD, standard deviation; ER, oestrogen receptor; PR, progesterone receptor; HER2, human epidermal growth factor receptor 2; HR, hormone receptor; RD, residual disease; pCR, pathologic complete response.

### 3.2 Diagnostic accuracy of surgical staging stratified by axillary disease extent on baseline ^18^F-FDG PET/CT

An overview of the diagnostic accuracy of surgical staging procedures for detection of axillary lymph nodes with residual disease stratified by axillary disease extent on baseline ^18^F-FDG PET/CT is displayed in Table 2. For the SLNB, MARI, and RISAS-procedure, the FNR was lower and the NPV higher (although not significantly) in patients with limited compared to advanced axillary disease on baseline ^18^F-FDG PET/CT.

**Table 2:** Diagnostic accuracy of SLNB, MARI, and RISAS-procedure stratified by axillary disease extent on baseline^18^F-FDG PET/CT.

	Overall		Hypermetabolic ALNMs on^18^F-FDG PET/CT				
			1-3		≥4		*P* value
**FNR**							
SLNB	22/110	(20.0)	12/73	(16.4)	10/37	(27.0)	.213
MARI	10/125	(8.0)	5/74	(6.8)	5/51	(9.8)	.739
RISAS	5/130	(3.8)	1/79	(1.3)	4/51	(7.8)	.077
**NPV**							
SLNB	50/72	(69.4)	33/45	(73.3)	17/27	(63.0)	.431
MARI	52/62	(83.9)	35/40	(87.5)	17/22	(77.3)	.307
RISAS	55/60	(91.7)	37/38	(97.4)	18/22	(81.8)	.056

Values in parentheses are percentages. ALNMs, axillary lymph node metastases; FDG, fluorodeoxyglucose; FNR, false negative rate; NPV, negative predictive value; SLNB, sentinel lymph node biopsy; MARI, marking the axilla with radioactive iodine seeds; RISAS, radioactive iodine seed localization in the axilla with sentinel lymph node biopsy. P-values concern comparisons between limited and advanced baseline axillary disease, given for each surgical staging procedure.

### 3.3 Residual disease at surgical staging and probability of remaining positive lymph nodes at cALND

Table 3 presents the probability that patients, who had residual disease after NST detected and removed at SLNB, MARI or RISAS-procedure, still had remaining positive lymph nodes at cALND. The results are stratified by axillary disease extent on baseline ^18^F-FDG PET/CT. For all three surgical staging procedures, the probability of remaining positive lymph nodes at cALND was lower in patients with limited compared to advanced baseline axillary disease (significant for MARI and RISAS-procedure). Overall, the risk of remaining positive lymph nodes exceeded 85% (88.4% to 100%), except for the group of patients with limited baseline axillary disease who had undergone the RISAS-procedure. In this group, the probability of remaining positive lymph nodes was 44.9% (35/78) and significantly lower than in the group with advanced baseline axillary disease (91.5% (43/47), p < .001). A mean (SD) of 1.9 (1.1) lymph nodes was removed with the RISAS-procedure (median [range], 2 [1–8] lymph nodes).

**Table 3:** Probability of remaining positive lymph nodes at cALND in patients with residual disease detected and removed at surgical staging, stratified by axillary disease extent on baseline^18^F-FDG PET/CT.

	Remaining positive lymph nodes at cALND						
	Overall		Hypermetabolic ALNMs on^18^F-FDG PET/CT				*P* value
			1-3		≥4		
SLNB	84/88	(95.5)	57/61	(93.4)	27/27	(100.0)	.308
MARI	107/115	(94.7)	61/69	(88.4)	46/46	(100.0)	**.021**
RISAS	78/125	(62.4)	35/78	(44.9)	43/47	(91.5)	**<.001**

Values in parentheses are percentages. cALND, completion axillary lymph node dissection; ALNMs, axillary lymph node metastases; FDG, fluorodeoxyglucose; SLNB, sentinel lymph node biopsy; MARI, marking the axilla with radioactive iodine seeds; RISAS, radioactive iodine seed localization in the axilla with sentinel lymph node biopsy.

P-values concern comparisons between limited and advanced baseline axillary disease, given for each surgical staging procedure.

## 4 Discussion

This unique retrospective study of the prospective multicentre noninferiority RISAS trial demonstrated that in all three surgical staging procedures, the FNR was lower and NPV higher in patients with limited (1-3 hypermetabolic ALNMs) compared to advanced (≥4 hypermetabolic ALNMs) axillary disease on baseline ^18^F-FDG PET/CT, although the differences were not statistically significant. Moreover, in case surgical staging detected and removed residual disease, the risk of remaining positive lymph nodes at cALND was high for all three procedures, ranging from 44.9% to 100%.

The combined approach of both SLNB and targeted lymph node biopsy is the most accurate method for surgical staging after NST in cN + breast cancer [4,7,10]. In this study, the FNR of the RISAS-procedure was as low as 1.3% in patients with limited baseline axillary disease. In patients with advanced baseline axillary disease, the FNR of the RISAS-procedure increased to 7.8%. Accordingly, high diagnostic accuracy of less invasive surgical staging procedures is essential, as the outcome of these procedures guide adjuvant axillary treatment. In case of axillary pCR, adjuvant treatment may not be required. The recent NSABP B-51/RTOG 1304 (NCT01872975) trial showed that omitting adjuvant axillary radiotherapy (ART) in cN1 patients with axillary pCR following NST did not comprise oncologic outcomes [20]. Notably, approximately half of the patients in this trial underwent ALND for axillary staging. The ongoing ATNEC (NCT04109079) trial is expected to provide further clarity, as it randomises cN1 patients with axillary pCR detected at SLNB to receive either ART, cALND, or no additional axillary treatment [21]. Importantly, these trials exclude patients with advanced baseline axillary disease. In combination with our finding of higher FNRs in patients with advanced baseline axillary disease, de-escalation approaches in this patient subgroup should be performed with caution.

In case of residual disease, adjuvant ART and/or cALND is considered necessary [11]. Moreover, adjuvant systemic treatment can be indicated with prognostic value in case of residual disease according to Create-X (NCT00130533) and KATHERINE (NCT01772472) trials [22,23]. Even in case of axillary pCR, patients with residual breast disease are still candidates for adjuvant systemic therapy and vice versa [24]. Van Loevezijn et al. recently published the 3-year follow up data of a protocol that combined axillary disease extent on baseline ^18^F-FDG PET/CT with the outcome of the MARI procedure in cN^+^ patients after NST [25]. In this protocol, cALND was omitted in 217/272 patients (80%) with a three-year probability of axillary recurrence free survival of 98% (95%-CI: 96.0-100.0). Of the five patients with axillary recurrence, all had limited (1-3 hypermetabolic ALNMs) baseline axillary disease. Four of the five patients (all with triple negative breast cancer) had residual disease demonstrated with the MARI procedure and underwent adjuvant ART without cALND [25]. In a recently published paper by Schipper et al. the 3-year follow-up results of a cohort of 199 cN + patients treated with a similar protocol were presented [26]. The 3-year probability of axillary recurrence free survival was also 98% and all four axillary recurrences occurred in triple negative breast cancer of whom 3 patients had adjuvant ART without cALND [26]. In current clinical practice, there was a trend towards replacing cALND with adjuvant ART in cN + patients with residual disease at surgical staging after NST. Although plausible, up to date it remains unclear whether adjuvant ART alone is sufficient to treat (therapy-resistant) residual disease in unresected axillary lymph nodes [11,27,28].

To offer insights into optimal adjuvant axillary treatment protocols in case of residual disease at less invasive surgical staging after NST, various clinical trials are currently ongoing. The Alliance A011202 (NST01901094) and TAXIS (NST03513614) trials investigate whether adjuvant ART can safely replace cALND in case of residual disease at less invasive surgical staging in cN+ patients treated with NST [29,30]. The results of these trials will guide adjuvant axillary treatment protocols that will optimize long-term prognosis and prevent both under- and overtreatment in cN+ patients with residual disease at less invasive surgical staging.

To guide decision making on the need for adjuvant axillary treatment in case of residual disease at less invasive surgical staging, it is relevant to assess the risk of (therapy-resistant) residual disease in unresected axillary lymph nodes. A study by Cabıoğlu et al. reported that the risk of remaining positive lymph nodes at cALND was lower in patients with only one metastatic axillary lymph node in their TAD specimen (27.6%) compared to patients with >1 metastatic axillary lymph nodes in their TAD specimen (54.8%) [31]. Along the same line, the current study found that in patients with residual disease at the RISAS-procedure, the probability of remaining positive lymph nodes at cALND was lower in patients with limited (44.9%) compared to advanced (91.5%) baseline axillary disease. In clinical practice, this information may be helpful to decide upon the need for (the extent of) adjuvant axillary treatment.

Importantly, our results do not suggest that axillary de-escalation should be restricted to patients with limited baseline axillary disease. A previous study from our study group showed that axillary disease extent on baseline ^18^F-FDG PET/CT was not a significant predictor of axillary pCR, indicating that having advanced baseline axillary disease does not rule out the probability of achieving axillary pCR and therefore allowing potential de-escalation following NST [32]. Accordingly, this study showed that stratification by baseline axillary disease can provide valuable insights for interpreting the outcome of less invasive surgical staging and the probability of remaining positive lymph nodes at cALND. As recent and ongoing de-escalation trials primarily categorize patients as cN1-3 according to the primary tumour, lymph node, and metastasis (TNM) classification of the American Joint Commission of Cancer [33], the results of this study may provide an argument to take the number of suspicious lymph nodes at baseline into consideration rather than relying solely on cN categories according to the TNM classification.

A strength of this study is that, although the RISAS-procedure is the most accurate less invasive surgical staging procedure compared to both standalone SLNB and MARI [10], all three surgical staging-procedures were correlated with axillary disease on baseline ^18^F-FDG PET/CT. This remains clinically relevant, as both standalone SLNB and MARI are widely used in clinical practice [34].

However, this study has some limitations. First, baseline axillary disease extent was only assessed on ^18^F-FDG PET/CT. Baseline ^18^F-FDG PET/CT imaging is accurate for regional and distant disease dissemination and has a positive predictive value of 98% in detecting ALNMs [35]. However, the results from this study are not generalizable to settings wherein other imaging modalities, including axillary ultrasound or breast MRI, are used at baseline for evaluation of the number of ALNMs. Second, central to this study was the comparison between two subgroups of patients (116 with limited and 69 with advanced axillary disease on baseline ^18^F-FDG PET/CT). The differences in FNR and NPV of the three surgical staging procedures between these two subgroups could be potentially clinically relevant but were not statistically significant in our study population. An explanation is that given the relatively low number of patients in both subgroups the power to detect significant differences between the groups was limited. Third, the data were not stratified by oestrogen (ER)-status. Although ER-status is known to influence the diagnostic accuracy of ^18^F-FDG PET/CT, with lower sensitivity generally observed in ER-positive tumours [36], stratification was not feasible due to the limited number of patients per subgroup. Despite this limitation, ^18^F-FDG PET/CT remains widely used in clinical practice across all breast cancer subtypes. Interestingly, other tumour specific tracers, such as fibroblast activation protein inhibitor (FAPI) [37], may hold potential future implications for subtype-specific imaging.

To conclude, the diagnostic accuracy of the SLNB, MARI, and RISAS-procedure was higher in patients with limited compared to advanced axillary disease on baseline ^18^F-FDG PET/CT. Moreover, when the SLNB, MARI, and RISAS-procedure detected and removed residual disease, the probability of remaining positive lymph nodes at cALND was high, ranging from 44.9% to 100% dependent on the type of staging-procedure and extent of axillary disease on baseline ^18^F-FDG PET/CT. Information on the probability of remaining positive lymph nodes in case surgical staging detected and removed residual disease may be relevant to guide further adjuvant axillary treatment for cN + patients treated with NST.

## CRediT authorship contribution statement

**Cornelis M. de Mooij:** Writing – review & editing, Writing – original draft, Visualization, Validation, Resources, Methodology, Investigation, Formal analysis, Data curation, Conceptualization. **Janine M. Simons:** Writing – review & editing, Writing – original draft, Resources, Methodology, Investigation, Data curation, Conceptualization. **Florien J.G. van Amstel:** Writing – review & editing, Writing – original draft, Visualization, Validation, Resources, Methodology, Investigation, Formal analysis, Conceptualization. **Cristina Mitea:** Writing – review & editing, Resources. **Paul J. van Diest:** Writing – review & editing, Resources. **Patty J. Nelemans:** Writing – review & editing, Resources, Methodology, Conceptualization. **Felix M. Mottaghy:** Writing – review & editing, Resources. **Carmen C. van der Pol:** Writing – review & editing, Resources. **Ernest J.T. Luiten:** Writing – review & editing, Resources. **Linetta B. Koppert:** Writing – review & editing, Resources. **Marjolein L. Smidt:** Writing – review & editing, Visualization, Resources, Methodology, Investigation, Conceptualization. **Thiemo J.A. van Nijnatten:** Writing – review & editing, Visualization, Supervision, Resources, Methodology, Investigation, Conceptualization.

## Data availability statement

Data and code for the final analyses are available from the corresponding author upon reasonable request.

## Ethical approval

The local medical ethics review committees of all participating institutions of the RISAS trial waived the necessity to acquire informed consent due to the retrospective study design.

## Sources of funding

Thiemo J.A. van Nijnatten and Florien J.G. van Amstel received funding for this study by the Dutch Cancer Society (REFINE-trial; project 14055). None of the other authors received support from any organization for the submitted work.

## Declaration of competing interest

The authors declare the following financial interests/personal relationships which may be considered as potential competing interests: Felix M. Mottaghy reports a relationship with GE Precision Healthcare LLC, NanoMab Technology Ltd., Radiopharm Ltd., and Siemens Healthcare that includes: funding grants. Felix M. Mottaghy reports a relationship with Advanced Accelerator Applications (AAA) GmbH/Novartis, CURIUMTM, NanoMab Technology Ltd., and Telix Pharmaceuticals that includes: consulting or advisory. Marjolein L. Smidt reports a relationship with Servier, Pharma, Nutricia and Illumina that includes: funding grants. Thiemo J.A. van Nijnatten reports a relationship with Bayer and GE Healthcare that includes: funding grants. Thiemo J.A. van Nijnatten reports a relationship with Screenpoint Medical that includes: consulting or advisory. If there are other authors, they declare that they have no known competing financial interests or personal relationships that could have appeared to influence the work reported in this paper.
